# Cortical oscillations and event‐related brain potentials during the preparation and execution of deceptive behavior

**DOI:** 10.1111/psyp.14695

**Published:** 2024-09-28

**Authors:** Robert Schnuerch, Jonas Schmuck, Henning Gibbons

**Affiliations:** ^1^ Department of Psychology University of Bonn Bonn Germany

**Keywords:** alpha suppression, CNV, cognitive control, deception, LPC, midfrontal theta, P2, P3

## Abstract

Deception often occurs in response to a preceding cue (e.g., a precarious question) alerting us about the need to subsequently lie. Here, we simulate this process by adapting a previously established paradigm of intentionally false responding, now instructing participants about the need for deception (vs. truthful responses) by means of a simple cue occurring before each response‐relevant target. We analyzed event‐related brain potentials (ERPs) as well as cortical oscillations recorded from the scalp. In an experimental study (*N* = 44), we show that a cue signaling the need for deception involves increased attentional selection (P2, P3a, P3b). Moreover, in the period following the cue and leading up to the target, ERP and oscillatory signatures of anticipation and preparation (Contingent Negative Variation, alpha suppression) were found to be increased during trials requiring a deceptive as compared to a truthful response. Additionally, we replicated earlier findings that target processing involves enhanced motivated attention toward words requiring a deceptive response (LPC). Moreover, a signature of integration effort and semantic inhibition (N400) was observed to be larger for words to which responses have to be intentionally false as compared to those to which responses must be truthful. Our findings support the view of the involvement of a series of basic cognitive processes (especially attention and cognitive control) when responses are deliberately wrong instead of right. Moreover, preceding cues signaling the subsequent need for lying already elicit attentional and preparatory mechanisms facilitating the cognitive operations necessary for later successful lying.

## Abstract 2

Using a straightforward experimental approach involving instructed false responding, we used event‐related brain potentials and analyses of cortical oscillatory activity to demonstrate increased executive control and attention during the processing of stimuli requiring deceptive (as compared to truthful) responses. We valuably expanded this by showing that preceding cues indicating the need for subsequent deception elicit additional processes of attention, anticipation, and preparation in the foreperiod leading up to the stimulus requiring a lie.

## 1 INTRODUCTION

Understanding the principles of deception is immensely important for at least two reasons: First, lying is an integral and perhaps indispensable aspect of social functioning (Saxe, 1991). Second, untruthfulness is a major challenge for law enforcement and the judiciary (Volbert & Banse, 2014). Not much surprisingly, a multitude of basic and applied studies have sought to identify the psychological and neurobiological foundations of deception (for review, see Abe, 2009; Levine, 2022). In addition to affective processes involved in deception (e.g., Abe et al., 2007; Porter et al., 2012), a series of cognitive mechanisms have been theorized and empirically shown to contribute to untruthful behavior: In order to successfully deceive, we have to (a) retrieve the truthful response and maintain it in working memory; (b) inhibit this truthful response; and (c) switch between two opposing tasks, that is, truthful and untruthful responding (Suchotzki et al., 2017).

To discern the contribution of basic cognitive functions to the overall process of deceptive behavior, neurophysiological methods are used in highly controlled laboratory experiments. Especially electroencephalography (EEG) and, based thereupon, event‐related brain potentials (ERPs) are well‐suited for this endeavor, seeing as they track the electrical signatures of neurocognitive operations with millisecond precision. Importantly, many of these signatures have been studied extensively and their functional significance (i.e., which cognitive process they reflect) has often been established (Luck, 2005).

### 1.1 ERP findings on the neurocognition of deception

To understand the neurocognitive principles of lying, a typical approach is to employ experimental paradigms involving intentional false responding. That is, participants are explicitly required to respond to an imperative stimulus (a target; often a single word or a question) truthfully on some trials, while they are asked to intentionally respond to targets untruthfully on others. While this clearly excludes motivational and affective aspects of real‐life lying (e.g., willingly opting to lie in order to achieve a goal), it maintains and even isolates its key cognitive components. Therefore, in spite of the somewhat artificial and restricted nature of these experimental protocols, such as the Differentiation of Deception Paradigm (Furedy et al., 1988) or the Sheffield Lie Test (Spence et al., 2001), they are valuable approaches to investigate the neurocognition of lying. Typically, the instruction to respond deceptively is delivered to participants by showing the target stimulus or the assignment of responses to buttons, which is shown below the target, in a certain color (see, e.g., Suchotzki & Gamer, 2018). Alternatively, one set of stimuli is pre‐assigned as those to which responses have to be deceptive and another set of stimuli as those to which responses must be truthful (Furedy et al., 1988). Either way, upon the appearance of the target, participants realize whether or not they have to respond truthfully on a given trial.

What varies in such experimental investigations is the ratio of trials requiring deceptive responses as compared to trials requiring truthful responses. Matching the numbers is obviously useful to eliminate any confound between veracity and likelihood (Spence et al., 2001). Using fewer deception trials than truth trials, however, allows the experimental setup to reflect the fact that, in everyday life, events requiring people to lie are far less frequent than those allowing them to respond truthfully (Verschuere et al., 2011). In both variants, physiological responses during the processing of the imperative targets are measured, and targets requiring a truthful response are contrasted with those requiring a deceptive response (Furedy et al., 1988, 1991).

For targets requiring deceptive as compared to truthful responses, previous studies have demonstrated increases in electrophysiological signatures indicating cognitive control in terms of conflict monitoring and resolution (mediofrontal negativity, MFN, and/or N2; see, e.g., Hein & Leue, 2021; Sai et al., 2023; Scheuble & Beauducel, 2020), attentional orienting (frontocentral P3a; see, e.g., Suchotzki et al., 2015), and sustained attention (posterior P3b; see Gibbons et al., 2018). One might argue that increased attention to targets requiring deceptive responses might be contingent upon the rarity of such events; however, it should be noted that this neatly reflects the infrequence of lies in real life and the natural involvement of typical neurophysiological responses to rare, task‐relevant stimuli (see Polich, 2007).

### 1.2 The sequential structure of lying

Interestingly, the typical experimental setups, as outlined above, have not yet covered a central aspect of deception: Many lies are not told proactively in response to very specific, pre‐determined stimuli (as in the Differentiation of Deception Paradigm; see Furedy et al., 1988) or triggered as late as upon the appearance of the specific stimulus to which one has to respond (as in the Sheffield Lie Test; see Spence et al., 2001). Situations resulting in lies often involve a preceding cue indicating the need to subsequently resort to a lie. Most lies are set off by social interactions, in which deceptive behavior represents a useful strategy of evading negative consequences (Saxe, 1991), such as by answering questions in a socially pleasing way. Seeing as humans frequently employ lies to maintain or strengthen social bonds (see DePaulo et al., 1996), we are likely to pick up the smallest of cues indicating the need to avoid truthful responses. That is, verbal interactions can involve sequences of cues (as to whether we should lie) and targets (to which we respond truthfully or deceptively). An example of this sequential nature is interrogations or interviews, in which law enforcement officials pose a question that—from the interviewee's perspective—serves as a cue that the subsequent response needs to be deceptive. For example, the interrogator might start a question saying “Last Friday at 10 p.m.…” (which already serves as a cue to the culprit that they must lie since this refers to the time of the crime) “…were you at home?” (which is the target to which the participant responds). Note that the first part of the question conveys the need for subsequent lying, even though the culprit does not yet know *how* to lie: The ending of the sentence might require an untruthful affirmation (as in the above example) or an untruthful negation (if the question ends in a mention of the location of the crime). This basic structure of an indicator of the upcoming need to deceive (cue) and a subsequent imperative stimulus (target) is likely to involve additional neurocognitive mechanisms that should clearly contribute to lying, but have not been investigated thus far.

A cue indicating the need for deception should attract attention; that is, it should stand out from other, more common stimuli that do not signal the somewhat rare need for lying (Verschuere & Shalvi, 2014). Therefore, similar to imperative stimuli that themselves signal the need for lying, such cues should lead to increased early and late attentional selection and orienting. This should be evident in the ERP by enhanced amplitudes of the P2 and P3a components during deception cues as compared to truth cues (see, e.g., Gibbons et al., 2018; Gupta et al., 2019; Nieuwenhuis et al., 2011; Polich, 2007). Moreover, deception cues should engage later, sustained attentional resources more strongly than cues indicating the need for the more frequent, default behavior (i.e., responding truthfully). Applying previous, more general findings on the allocation of sustained attention (see, e.g., Verleger, 2020) to the context of deception, an increased P3b amplitude during the processing of deception cues as compared to truth cues seems likely.

Additionally, the time between the cue signaling the (rare and relevant) need for lying and the actual appearance of the imperative stimulus to which this lie has to be told should involve processes of anticipation and preparation. That is, the high level of cognitive demand of lying upon the target's appearance (Suchotzki et al., 2017) should lead to greater anticipatory and preparatory activity in the foreperiod between learning about the need to lie and actually perceiving the imperative stimulus about which one has to lie. A neurophysiological marker of the perceptual, attentional, cognitive, and motor processes involved during such foreperiods (between cue and target) is the Contingent Negative Variation (CNV; Walter et al., 1964). It is assumed to reflect anticipatory and preparatory processes (see, e.g., Anatürk & Jentzsch, 2015; De Kleine & Van Der Lubbe, 2011; Hackley et al., 2014; Kóbor et al., 2021; Pauletti et al., 2014; Stadler et al., 2006; Wöstmann et al., 2015). Relevant stimuli (e.g., Getzmann et al., 2016), rare events (e.g., Bauer et al., 1992), and highly demanding tasks (e.g., Wild‐Wall et al., 2007) involve increases in preceding CNV amplitude. Therefore, the amplitude of the CNV should be increased following cues indicating the need for lying as compared to cues indicating the need for truthful responding.

### 1.3 Analyzing cortical oscillations

Interestingly, EEG recordings can be used to track preparatory and anticipatory processes not only by means of EPRs, but also by monitoring cortical oscillations. Anticipating sensory events has previously been demonstrated to elicit the suppression of alpha power (i.e., oscillatory activity in the 8–13 Hz frequency band) in the specific areas involved in processing the upcoming event. The suppression of alpha oscillations appears to reflect increased cortical excitability in task‐relevant neurons in order to facilitate subsequent processing (Noah et al., 2020). For example, when anticipating a visual stimulus demanding an analysis of its spatial motion, alpha oscillations were found to be suppressed in the dorsal visual stream in the period before the onset of the stimulus. Anticipating a stimulus that needs to be processed in terms of its color led to suppressed alpha oscillations in the ventral visual stream (Snyder & Foxe, 2010). This shows that the brain structures that need to be recruited to best process the upcoming stimulus are prevented from returning to an “idle mode” (alpha oscillations), such that their readiness or excitability is higher when the predicted stimulus appears. Applying this to the process of deceptive (i.e., intentionally false) responding following a cue, one should expect to observe preparatory alpha suppression in brain structures heavily involved in the processing of the upcoming target stimulus. Which brain areas should be involved is slightly less straightforward to determine. One possibility for target *words* (as in previous studies; see, e.g., Gibbons et al., 2018) could be structures involved in the representation of word meaning (Pulvermüller, 2001). Another possibility could be neural processes involved in executive control, such as those observed in the anterior cingulate cortex (ACC) or the prefrontal cortex (PFC; for an overview, see Salehinejad et al., 2021). Moreover, brain structures involved in controlled, purposeful actions, such as the frontoparietal network assumed to underlie intentional behavior (e.g., Brass & Haggard, 2008; Forstmann et al., 2006; Haggard, 2005; Wisniewski et al., 2016), could be candidates for preparatory alpha suppression between lying cues and imperative targets.

Moreover, cognitive control, a key component of successful lying (Suchotzki et al., 2015), can also be tracked by analyzing cortical oscillations. Previous studies have repeatedly demonstrated that target words to which false (as compared to truthful) responses have to be given intentionally in deception paradigms involve increased ERPs signatures of executive control processes such as conflict detection and resolution (Gibbons et al., 2018; Hu et al., 2012; Leue et al., 2012; Scheuble & Beauducel, 2020; Suchotzki et al., 2015). EEG‐derived cortical oscillations also provide a highly sensitive, valid marker of cognitive control, namely midfrontal activity in the theta band (4–8 Hz; for review, see Cavanagh & Frank, 2014). Therefore, it stands to reason that words requiring deceptive responses should entail stronger frontal midline theta oscillatory activity than words requiring a truthful response.

### 1.4 The present study

In the present study, we tested the above predictions in a simple experimental task modeled after previous investigations into the neurocognition of lying (see, e.g., Gibbons et al., 2018; Hu et al., 2011, 2012; Suchotzki et al., 2015). In our novel, straightforward design, the target words to which participants were asked to respond truthfully or with a lie were preceded by a cue indicating the need for lying or truth‐telling. Following the setup of the Sheffield Lie Test, we used two different colors to indicate with a simple cue (fixation cross) what type of behavior (lie vs. truth) was expected in response to the upcoming target (Spence et al., 2001). However, we maintained the approach of reflecting the rarity of lies (Verschuere & Shalvi, 2014) by asking participants to respond deceptively far less frequently than asking them to tell the truth (for an extensive discussion of this approach, see Gibbons et al., 2018). Participants responded in a binary fashion to simple target words by categorizing them as items of clothing or items of furniture.

Based upon previous behavioral, psychophysiological, and neuroimaging studies, we predicted that participants should respond more slowly and with more errors to targets requiring a lie than to those requiring a truthful response (Suchotzki et al., 2017). Also, we expected to find the abovementioned amplitude increases for the P2, P3a, and P3b components of the ERP time‐locked to the onset of the response‐guiding cue. Additionally, we expected an increased CNV following the (lying as compared to truth‐telling) cue in the foreperiod leading up to the onset of the target. Likewise, we expected to find alpha suppression (i.e., reduced alpha‐band activity) in this foreperiod; the neural structures involved in this effect were somewhat unclear, which is why we did not specify any region of interest (ROI). Finally, we expected to replicate previous findings on ERP effects for the actual target stimuli that require deceptive as compared to truthful responses (Gibbons et al., 2018; Hein & Leue, 2021; Hu et al., 2011, 2012; Johnson Jr. et al., 2005, 2008; Koeckritz et al., 2019; Leue et al., 2012; Leue & Beauducel, 2019; Meijer et al., 2014; Scheuble & Beauducel, 2020; Suchotzki et al., 2015). That is, we should find increased neural signatures of conflict monitoring (MFN) and sustained attention (LPC) for targets requiring deceptive as compared to those requiring truthful responses. Additionally, we tested whether frontal theta oscillations (4–8 Hz), which reflect conflict processing on the level of neural oscillations, would also be increased during targets requiring a deceptive response.

## 2 METHOD

### 2.1 Participants

A total of 47 undergraduate students at the University of Bonn (18 male) volunteered in exchange for partial course credit or monetary compensation (8 euros per hour). Due to technical difficulties during recording, three participants had to be excluded from analysis, resulting in a total of 44 participants. Their age ranged from 19 to 32 years (*M* = 22.5; SD = 2.9). All of them were fluent in German or native speakers, had normal or corrected vision, reported no history of neurological disorders, and gave written informed consent. The study was conducted in accordance with the ethical guidelines of the German Psychological Association and approved by the local ethics committee (#21‐11‐29).

### 2.2 Stimuli and apparatus

**FIGURE 1:**
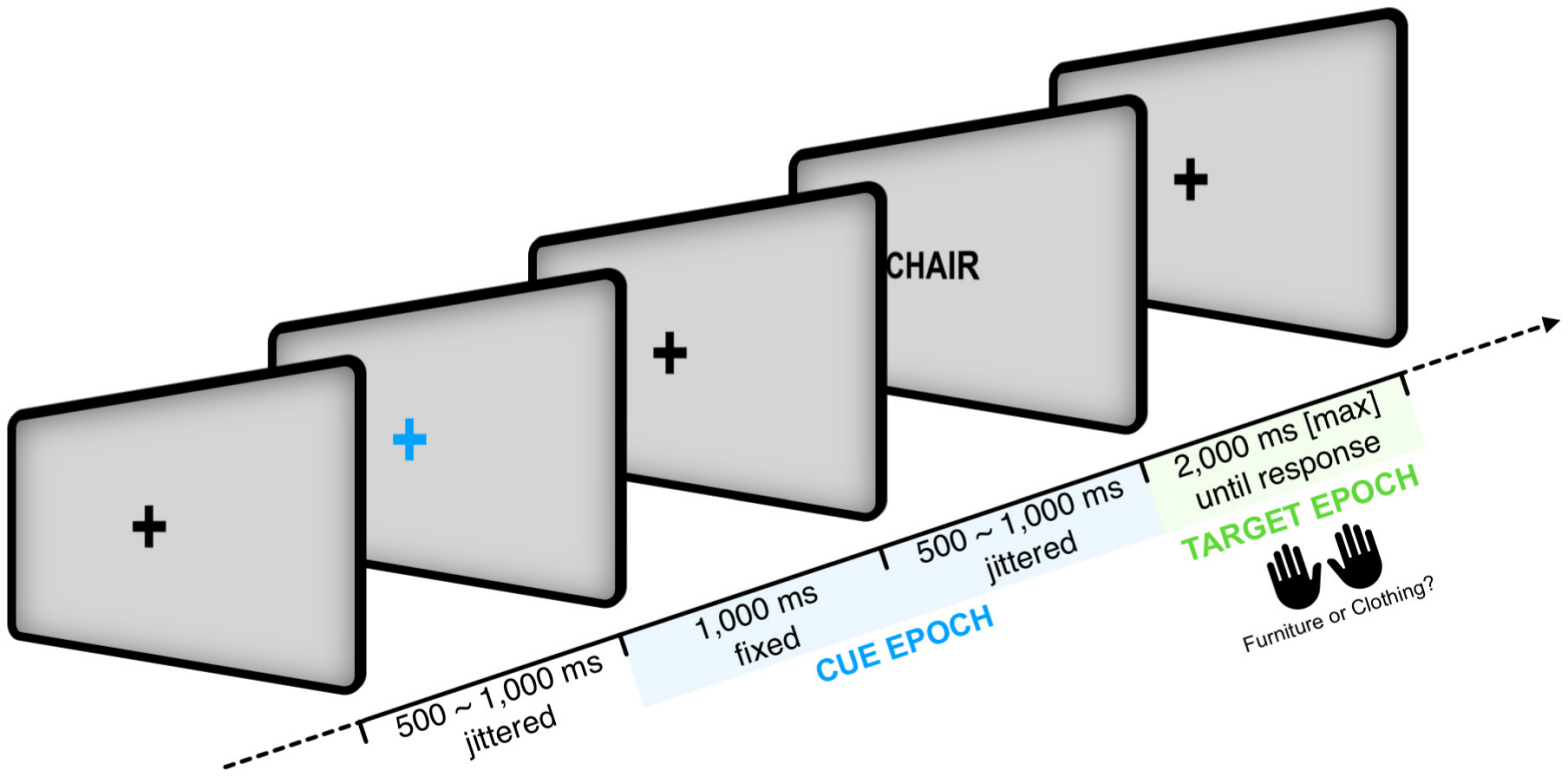
Overview of the structure of trials during the experiment. Note that the color change of the fixation cross (cue) indicates the need for a truthful versus an intentionally untruthful response. Color assignments and response mapping were counterbalanced across participants.

As cues indicating whether a truthful or a deceptive response had to be given in response to a subsequent target word, the otherwise white fixation cross turned yellow or blue. The assignment of colors to conditions (lie vs. truth) was counterbalanced across participants. Target stimuli were five German nouns from the category of furniture (Couch [couch], Liege [divan bed], Stuhl [chair], Regal [shelf], Tisch [table]) and five from the category of clothing (Anzug [suit], Kleid [dress], Jacke [jacket], Bluse [blouse], Mütze [cap]). All German nouns consisted of five letters each, and the two sets (furniture vs. clothing) were matched in terms of emotionality, imagery, relevance, and frequency (Hager & Hasselhorn, 1994; see also Gibbons et al., 2018). The cue symbols and the target words were shown on a black background in a bold monospace font (Courier New, 25 pt), placed at the center of a 23‐inch TFT display, thus measuring 0.5 cm (height) by 2.7 cm (width) on screen. All target words were shown in capital letters. All responses were given via the left and right control keys of a standard keyboard placed on the table in front of participants. The assignment of responses (furniture vs. clothing) to keys (left vs. right response key) was counterbalanced across participants (Figure 1).

### 2.3 Procedure

Upon arrival of the participants, EEG was prepared and participants were informed about the purpose of the study (semantic categorization of words with intentionally false vs. truthful responses based on a colored cross preceding each word). The experiment took place in a soundproof, dimly lit chamber, in which participants sat comfortably with their heads placed at a distance of approximately 80 cm from the screen.

Participants learned that their task would be to classify words as belonging to the category of furniture or clothing by binary button press (left vs. right response key; see Figure 1). However, the color of the fixation cross would indicate whether or not they should respond truthfully to the upcoming target word. Each trial consisted of a white fixation cross that was shown for 500–1000 ms at the center of the screen. Subsequently, the fixation cross turned either yellow or blue for 1000 ms, thus delivering the instruction regarding the truthfulness of the upcoming response. After that, the fixation cross was shown in white color for another 500–1000 ms. The subsequent target word was shown for a maximum of 2000 ms. Responses were given via button press; incorrect (regarding the instruction, thus not necessarily regarding the actual ground truth) responses were followed by an error feedback, as were late responses (button press later than 2000 ms after target onset).

The experiment consisted of 8 blocks containing 50 trials each, and participants could take self‐paced breaks between the blocks. Including 15 introductory practice trials (with full feedback for correct, incorrect, and late responses) that were not entered into analysis, the experiment comprised 415 trials. All words were presented in random order, with each word being shown 40 times. Of the 400 non‐practice trials, 320 (80%) contained truth cues (i.e., the cue assumed the color signaling the need to respond truthfully), while 80 (20%) contained lie cues, with both types of trials counterbalanced across blocks and words. The whole experiment lasted for about 30 minutes. Participants subsequently filled in questionnaires covering sociodemographic variables and handedness. Upon completion of the experiment, participants received course credit or payment.

### 2.4 Statistical power

Our main analyses involved the comparison of behavioral and electrophysiological mean values for the lie vs. the truth condition. We therefore used the software G*Power 3.1 (Faul et al., 2007) to evaluate the statistical power (1 – β) of repeated‐measures analyses of variance (ANOVAs) with two measurements. This revealed that for small to medium effects sizes (*f* = .2), our final sample size (*N* = 44) allowed us to achieve a power of .74; for medium‐sized effects (*f* = .25), statistical power was >.9. Therefore, our sample was sufficient to unveil substantial effects of veracity on behavioral and physiological measurements.

### 2.5 Frequentist and Bayesian analyses

In addition to frequentist analyses using *t*‐tests and ANOVAs, we computed the corresponding Bayesian repeated‐measures ANOVAs to further explore how probabilities for null hypothesis and alternative hypothesis differ. All analyses were conducted with the default Jeffreys–Zellner–Siow priors on the parameters and the a‐priori assumption that all models are equally likely. As recommended by Keysers et al. (2020), effects are reported as the Bayes factor (BF_incl_) for the inclusion of a particular effect across matched models. BF are usually interpreted as continuous measures of evidence, but we adopted the following labels for the strength of evidence as expressed by BF_incl_: 1 for no evidence, between 1 and 3 for anecdotal evidence, between 3 and 10 for moderate evidence, between 10 and 30 for strong evidence, between 30 and 100 for very strong evidence, and larger BFs for extreme evidence favoring the models including the particular effect. Conversely, 0.33–1.00, 0.10–0.33, 0.03–0.10, 0.01–0.03, and BF_incl_ <0.01 express increasing evidence in favor of the models without that particular effect (Jeffreys, 1961).

### 2.6 EEG recording and general preprocessing

EEG was recorded from 64 sintered Ag/AgCl electrodes placed according to the 10/10 system, using a digital 64‐channel BrainAmp system (Brain Products, Gilching, Germany), with four electrodes being used to record ocular motion and one electrode serving as reference during recording. The ground electrode was located close to AFz; FCz served as reference electrode during recording. Vertical and horizontal electrooculograms (EOG) were monitored from electrodes mounted below and above the right eye, and at the outer canthi, respectively. EEG was recorded at a sampling rate of 500 Hz, impedances of all electrodes were kept below 5 kΩ.

EEG data processing was performed using the Python module MNE (Gramfort et al., 2013), version 1.2.1 running on Python version 3.10.6. The continuous data were high‐pass filtered at 0.1 Hz (Acunzo et al., 2012; Tanner et al., 2015) using a Butterworth IIR filter (order 4) and low‐pass filtered at 30 Hz (order 4). Additionally, a notch filter was applied at 50 Hz. Bad channels were automatically detected using the Python module pyprep which implements the recommendations from the PREP Pipeline (Bigdely‐Shamlo et al., 2015). All channels that deviated either in terms of the bad‐by‐high‐frequency‐noise or the bad‐by‐deviation criteria (with *z* score >5) were interpolated using a spline interpolation procedure (min = 0; max = 9; mean = 2.07). Data were then re‐referenced to digitally linked mastoids.

To correct for eyeblink artifacts, an ICA (Picard algorithm; Ablin et al., 2018) was conducted on a separate dataset which was filtered with a high‐pass filter at 1 Hz and low‐pass filter at 30 Hz (Winkler et al., 2015). MNE's standard algorithm to detect problematic EOG data was used to automatically identify ICA components associated with ocular artifacts (as indicated by high correlations with ocular channels), using a threshold of *z* > 4. This resulted in the removal of 2.2 components on average (min = 1; max = 4). The ICA decomposition was then applied to the original dataset.

### 2.7 ERP preprocessing and analysis

For ERP analysis, continuous data were segmented into epochs of 1600 ms (−100; 1500 ms) relative to cue and target onset and baseline corrected to the 100‐ms pre‐stimulus interval. Segments containing amplitudes exceeding ±120 μV were rejected. On average, 2.2% of the cue trials and 10.72% of the target trials were rejected. A detailed overview of the number of artifact‐free trials in all relevant experimental conditions is provided in Table 1. For target trials, only those were included in all analyses on which participants responded correctly (as per the cue‐based instruction).

**TABLE 1:** Artifact‐free epochs in all relevant experimental conditions.

Event	Veracity	*M*	SD	Min	Max
Cue	Lie	78.55	2.01	71	80
	Truth	312.59	7.59	289	320
Target	Lie	72.20	5.21	53	79
	Truth	301.84	12.73	254	318

Abbreviations: M, mean; Max, maximum; Min, minimum; SD, standard deviation.

To identify the appropriate time windows and recording sites to assess the amplitudes of the ERP components of relevance for the present investigation, we used the collapsed‐localizer approach (Luck & Gaspelin, 2017). That is, ERPs were averaged across conditions and inspected regarding the to‐be‐expected components. Consequently, location and timing of the respective components were narrowed down without knowledge of where the experimental effect (i.e., the influence of veracity on amplitude) would be largest.

The grand‐grand average ERP during cue processing, collapsed across all experimental and control conditions, revealed marked components at the following recording sites and in the following time ranges: a posterior N2 (appropriately averaged across electrodes PO7, PO8, O1, O2) in the time window 190–240 ms; a frontocentral P2 (F1, Fz, F2, FC1, FCz, FC2, C1, Cz, C2) in the time window 160–220 ms; a frontocentral P3a (F1, Fz, F2, FC1, FCz, FC2, C1, Cz, C2) in the time window 260–340 ms; a centroparietal P3b (P1, Pz, P2, CP1, CPz, CP2, C1, Cz, C2) in the time window 400–600 ms; and a frontocentral CNV (FC1, FCz, FC2, C1, Cz, C2) in the time window 1000–1500 ms. Note that the P3a was largest at frontal electrodes, which is why our analysis focused on the abovementioned sites (see also Table 2). The P3a *effect* (i.e., the voltage difference between truth and lie trials) was seemingly largest at posterior sites (see Figure 4). However, the P3a effect is mostly masked by the almost concurrent P3b effect, which is located at posterior sites and far greater in voltage. In line with the literature on the P3a and with the fact that the actual component (rather than the difference wave) was observed at the to‐be‐expected frontal sites, P3a was quantified and analyzed at these electrodes.

**TABLE 2:** Overview of ERP components, their location, timing, and factors during analysis.

Event	Component	Position (averaged across electrodes)	Time window	Factors for analysis
Cue	N2	PO7, PO8, O1, O2	190–240	Control factor: *color assignment* (A vs. B) Control factor: *button assignment* (A vs. B)
	P2	F1, Fz, F2, FC1, FCz, FC2, C1, Cz, C2	160–220	
	P3a	F1, Fz, F2, FC1, FCz, FC2, C1, Cz, C2	260–340	
	P3b	P1, Pz, P2, CP1, CPz, CP2, C1, Cz, C2	400–600	
	CNV	FC1, FCz, FC2, C1, Cz, C2	1000–1500	
Target	N400	CPz, P1, Pz, P2, POz	380–480	
	LPC	FC1, FCz, FC2, C1, Cz, C2, CP1, CPz, CP2	650–1150	

*Note*: The experimental factor for all analyses was a repeated‐measures factor (veracity). Additional factors for control analyses were between‐subjects factors (color assignment and button assignment). For color assignment, A means that yellow signals lie, while blue signals truth; B means that yellow signals truth, while blue signals lie. For button assignment, A means that to categorize a target as furniture, the left button is used, while the right button is used to categorize targets as clothing; B means that responses are assigned to buttons the other way around.

The grand‐grand average ERP during target processing, collapsed across all experimental and control conditions, revealed components at the following recording sites and in the following time ranges: while no typical MFN was observed, a slightly later, centroparietal N400 (CPz, P1, Pz, P2, POz) in the time window 380–480 ms; and an LPC, distributed widely from frontocentral to centroparietal sites (FC1, FCz, FC2, C1, Cz, C2, CP1, CPz, CP2) in the time window 650–1150 ms.

Based upon the previously determined time windows and electrode clusters, mean voltages for the separate ERP components (five during cue processing; two during target processing) were submitted to repeated‐measures ANOVAs involving a within‐subject factor Veracity (truth vs. lie) and between‐subjects factors Button Mapping (furniture left vs. furniture right) and Color Mapping (yellow for truth vs. yellow for lie). The between factors were included to control for potential effects of counterbalanced color and/or button assignments on the hypothesized experimental effects of veracity. Since separate hypotheses existed for all comparisons, no correction for multiple comparisons was necessary. It should be noted, though, that all of the significant effects reported below would still be significant if Bonferroni‐Holm corrections were applied (all *p*_c_ < .05).

### 2.8 Time‐frequency preprocessing and analysis

For time‐frequency analysis, we created longer epochs ranging from −1200 ms to 2000 ms for targets and −1200 to 2500 ms for cues to provide a time window which was long enough to analyze theta and alpha‐band oscillations, respectively. All epochs were downsampled to 250 Hz in order to increase computational speed. Cue and target epochs were first transformed into current‐source density (CSD) data (Kayser & Tenke, 2015). Following recommendations from the FLUX pipeline (Ferrante et al., 2022), epochs were decomposed into their time‐frequency representations of power using a (multi‐) taper approach with sliding time windows applied to the single trials (Tallon‐Baudry & Bertrand, 1999). Analyses were carried out in 40 logarithmically spaced steps ranging from 1 to 30 Hz while the number of cycles was set at half of the respective frequency. This resulted in a time‐window length of 500 ms. A single discrete prolate spheroidal (DPSS) taper was multiplied to each time window prior to calculating the discrete Fourier transform (time bandwidth = 2.0). The resulting power estimates per trial were subsequently averaged. The relative change in power (percentage mode) was then considered with respect to a baseline (−400 to −200 ms).

The Dynamics Imaging of Coherent Sources (DICS) beamformer (Gross et al., 2001) was computed on single‐subject level for the two conditions for cue and target epochs separately. To localize the modulations of alpha (cue epochs) and theta (target epochs) oscillatory brain activity, the transformation was applied to average‐referenced data in the same time and frequency window that was shown to reveal significant effects at the sensory level for the time‐frequency analysis. First, we defined the volume source space on the fsaverage template brain model provided by FreeSurfer and implemented in MNE (Dale et al., 1999; Fischl et al., 1999). Given the source space, we constructed a forward model by employing a three‐shell boundary element method (BEM) model (Hämäläinen et al., 1993). The BEM model uses the FreeSurfer meshes of the brain tissues (inner skull surface, outer skull surface, and scalp surface). Then, this was used to construct a volumetric forward model (5 mm grid) covering the full brain volume. The lead field matrix was calculated according to the head position with respect to the EEG sensor array.

Based upon the temporal dynamics of power peaks for alpha oscillations (8–13 Hz) during cue processing and theta oscillations (4–8 Hz) during target processing, the DICS beamformers were constructed as follows (separately for cue and target epochs): For cue epochs, beamformers were derived from the interval 500–1100 ms after stimulus onset as well as the in the −800 to −200 ms pre‐stimulus interval (baseline). For target epochs, beamformers were derived from the interval 400–900 ms after stimulus onset as well as the in the −500 to 0 ms pre‐stimulus interval (baseline). Alpha activity for cue epochs and theta activity for target epochs were assessed for the respective intervals through a spectral analysis where a multitaper frequency transformation (using one DPSS taper) was implemented to generate the cross spectral density (CSD) matrix. The combined CSD was used with the forward model to create a common spatial filter with regularization parameter as 5%. This was done by first estimating the rank of the data and spatially pre‐whitening the data using the covariance matrix from the baseline interval. The truncated pseudo‐inverse was then calculated based on the estimated rank (Jas et al., 2018). For each source, the orientation was optimized to maximize the power of the output. The spatial filter was then applied to each CSD to extract the source and its power. Finally, the beamformer was calculated by subtracting the two conditions (lie vs. truth) and baseline‐normalizing the difference by the pre‐stimulus data (van Vliet et al., 2018).

Statistical analysis of the time‐frequency maps was calculated using non‐parametric cluster‐based permutation tests (Maris & Oostenveld, 2007) to control for multiple comparisons. For the cue epochs, we focused on centroparietal sites revealing notable alpha power differences based on an inspection of the spatial distribution across the scalp (CP3, CP5, CP4, CP6, P3, P5, P4, P6). Based upon the temporal sequence of all trials, cue epochs were analyzed between 0 ms (cue onset) and 2000 ms. For target epochs, we focused on electrode FCz as it shows strong midfrontal theta oscillations during conflict processing (Cavanagh et al., 2012; Cohen & Cavanagh, 2011; Driel et al., 2012). The time window was 0 ms (target onset) to 1000 ms. For cues and targets separately, power differences between lie and truth trials were identified by means of cluster‐based permutation paired *t*‐tests, which were conducted on three‐dimensional data, that is, on all (subject, frequency, time) triplets.

The procedure for the cluster‐based paired *t* test analyses was as follows. Adjacent frequency‐bins and time‐points with *p*‐values below .001 were grouped into clusters. Cluster‐based statistics were computed as the sum of *t*‐values within a cluster. We then determined the significance probability of the cluster statistic by means of a permutation test. The permutation distribution was created by randomly sign flipping the power difference values and extracting the maximum cluster‐level statistic. We repeated this procedure 10,000 times to obtain a reference distribution of test statistics. The cluster *p*‐value was then obtained as the proportion of permutations above the observed cluster‐based statistic. The statistical threshold was set at 2.5% for the largest positive and negative clusters, providing a two‐tailed 5% alpha level of family‐wise error control for multiple comparison correction.

## 3 RESULTS

### 3.1 Behavior

**FIGURE 2:**
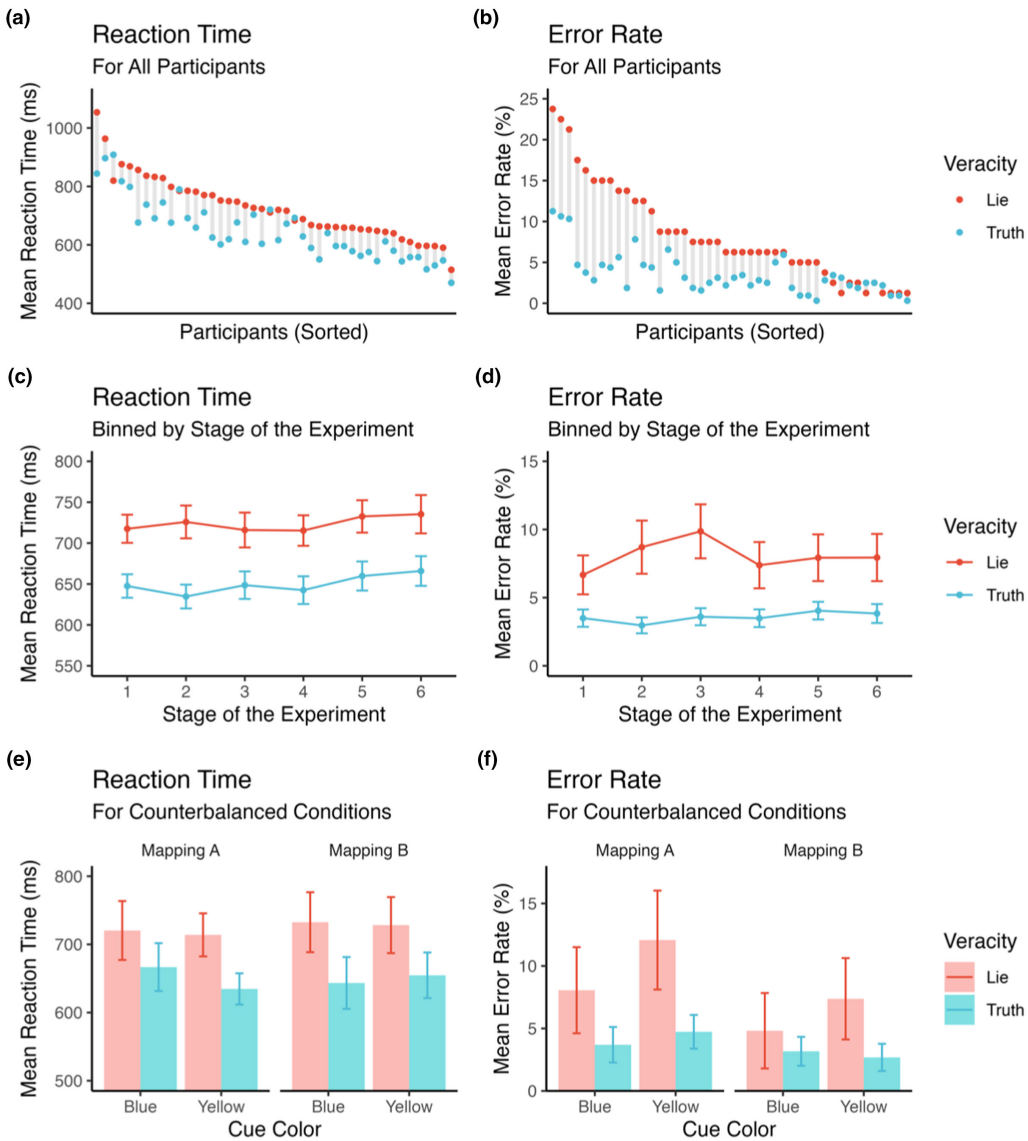
Behavioral effects. Reaction times and error rates were substantially larger for words requiring an untruthful as compared to a truthful response. This pattern was evident for almost all participants (a, b). Also, it occurred across the whole experiment, not systematically increasing or decreasing over time (c, d). While details of the experimental setup (color assignment and response mapping) slightly affected the effect of veracity on reaction times and error rates, these effects were robustly observed in all conditions (e, f). Errors include both incorrect responses and rare misses due to responses occurring late (i.e., not within 2000 ms following target onset).

All descriptive behavioral results are shown in Figure 2. A repeated‐measures ANOVA with reaction time (for correct responses) as dependent variable and within‐subject factor Veracity (truth vs. lie) revealed a substantial difference between the two experimental conditions (truth vs. lie trials) in terms of reaction time, with markedly slower responses in lie as compared to truth trials [*F*(1,43) = 91.13, *p* < .001, *η* ^2^_g_ = .12, BF_incl_ = 2.16^e+19^].

To additionally control for the potential (unwanted) influence of time‐on‐task, stimulus category, and the counterbalanced assignments of experimental parameters, a repeated‐measures ANOVA with reaction time (for correct responses) as dependent variable and within‐subject factors Veracity (truth vs. lie), Stage (first half vs. second half), and target category (furniture vs. clothing) as well as between‐subjects factors Button Mapping (furniture left vs. furniture right) and Color Mapping (yellow for truth vs. yellow for lie) was run. This analysis revealed that the main effect of Veracity was not further modulated by the mapping of responses to buttons, by the color mapping for lie vs. truth trials, by the stimulus category, or by the stage during the experiment, all *p*s > .385.

In analogous fashion, error rate was first submitted to a repeated‐measures ANOVA with reaction time (for correct responses) as dependent variable and within‐subject factor Veracity (truth vs. lie). This revealed a substantial difference between the two experimental conditions (truth vs. lie trials) in terms of error rate, with a higher percentage of errors in lie as compared to truth trials [*F*(1,43) = 53.50, *p* < .001, *η* ^2^_g_ = .22, BF_incl_ = 5.22^e+11^].

To control for the potential influence of time‐on‐task, stimulus category, and the counterbalanced assignments of experimental parameters, an repeated‐measures ANOVA with error rate as dependent variable and within‐subject factors Veracity (truth vs. lie), Stage (first half vs. second half), and target category (furniture vs. clothing) as well as between‐subjects factors Button Mapping (furniture left vs. furniture right) and Color Mapping (yellow for truth vs. yellow for lie) was run. This revealed that the main effect of Veracity was further modulated by Button Mapping [*F*(1,40) = 6.752, *p* = .013, *η* ^2^_g_ = .02], with a slightly stronger difference between error rates for lie vs. truth trials for the assignment of the response “furniture” to the left as compared to its assignment to the right button. Likewise, the effect of Veracity on error rates was modulated by Color Mapping [*F*(1,40) = 7.673, *p* = .008, *η* ^2^_g_ = .02], with a stronger difference between error rates for lie vs. truth trials when yellow cues signaled the need for deceptive responses (and blue signaled the need for truthful responses) as compared to yellow signaling the need for truthful responses (and blue signaling the need for deceptive responses). No further interactions were observed, with all interaction terms involving the factor Veracity yielding *p*s > .273.

### 3.2 Cue processing

#### 3.2.1 Exploratory ERP analyses (posterior N2)

**FIGURE 3:**
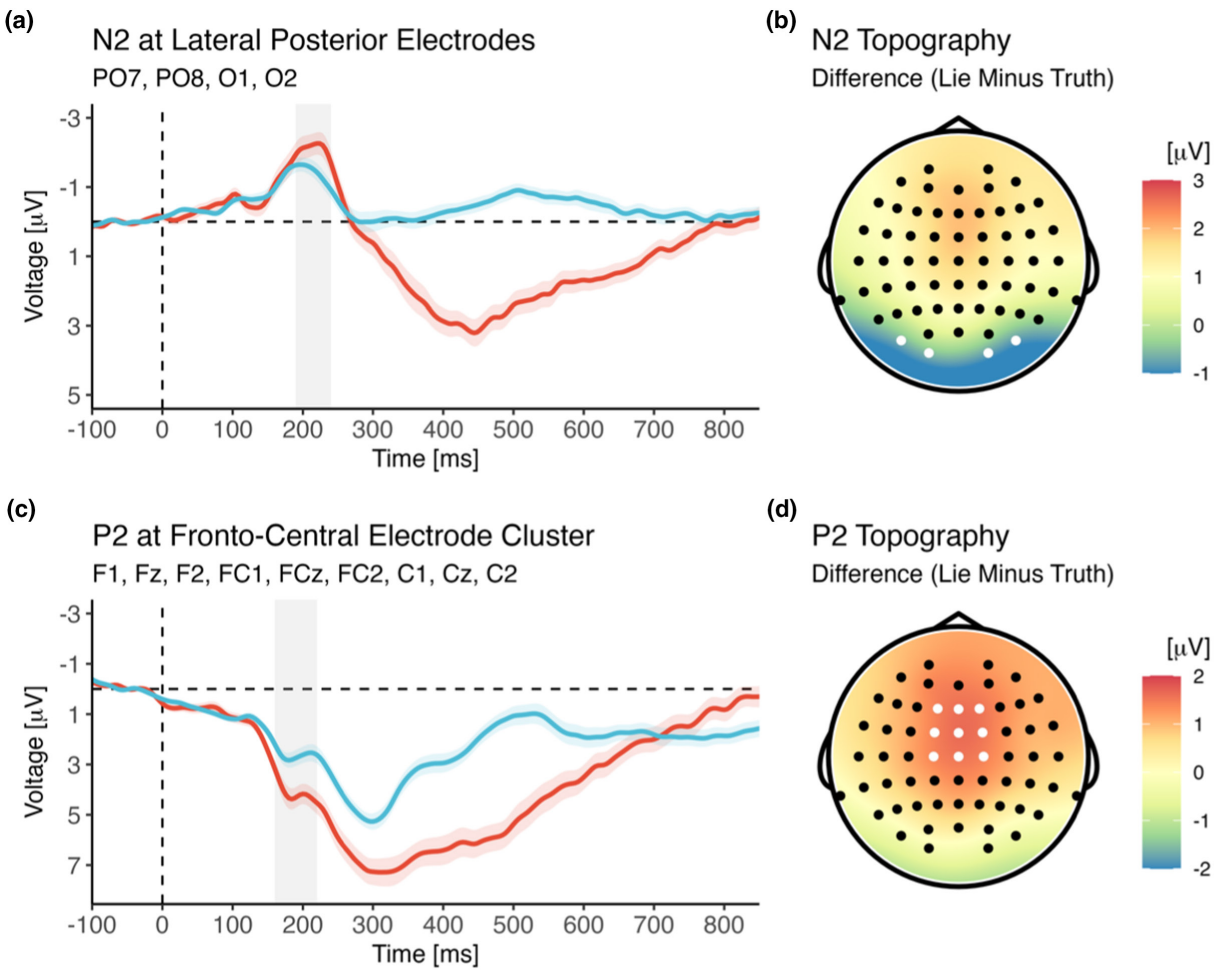
Effect of veracity on ERP amplitudes for early attentional components N2 (a, b) and P2 (c, d). The shaded area marks the time window selected for analysis (see main text for details).

While we did not make any predictions as to potential differences between lie and truth trials during early attentional stages of processing, inspection of the ERP revealed a marked difference between the two experimental conditions (lie vs. truth) at posterior electrodes for the N2 component (see Figure 3). To explore this finding thoroughly (despite the lack of predictions regarding its appearance), we submitted it to analyses analogous to those applied to the components for which hypotheses had been made (P2, P3a, P3b, CNV). An overview of all descriptive ERP results (i.e., mean amplitudes and respective standard errors) as a function of the relevant experimental factor Veracity (with two levels: lie vs. truth) is provided in Table 3.

**TABLE 3:** Mean ERP component amplitudes.

Epoch	Component	Experimental condition	
		Lie	Truth
Cue	N2	−2.11 (0.27)	−1.41 (0.20)
	P2	4.13 (0.35)	2.63 (0.27)
	P3a	7.05 (0.47)	4.80 (0.29)
	P3b	6.31 (0.41)	−0.29 (0.23)
	CNV	−2.02 (0.44)	1.01 (0.33)
Target	N400	2.80 (0.68)	5.05 (0.71)
	LPC	6.94 (0.67)	2.57 (0.49)

*Note*: All values are mean amplitudes (averaged across the time windows and recording sites listed in Table 2), with standard errors provided in parentheses.

Lie trials involved a larger posterior N2 than truth trials [main effect of Veracity; *F*(1,40) = 10.32, *p* = .003, *η* ^2^_g_ = .07], BF_incl_ = 2.91. This effect was further modulated by the assignment of colors to conditions [interaction of Veracity × Color Mapping; *F*(1,40) = 43.01, *p* < .001, *η* ^2^_g_ = .23, BF_incl_ = 7.51^e+5^]. Follow‐up ANOVAs revealed that the effect of Veracity was significant for yellow/truth and blue/lie [*F*(1,21) = 7.06, *p* = .015, *η* ^2^_g_ = .07, BF_incl_ = 3.61] as well as for yellow/lie and blue/truth [*F*(1,21) = 42.70, *p* < .001, *η* ^2^_g_ = .41, BF_incl_ = 1.14^e+5^]. It is evident, however, that the N2 effect of veracity was notably stronger when yellow indicated the need for lying and blue signaled the need for a truthful response. No further interactions involving the factor Veracity were observed (all *p*s > .731).

#### 3.2.2 Hypothesis‐driven ERP analyses (P2, P3a, P3b, CNV)

As expected, a strong P2 difference between lie and truth trials was observed at frontocentral electrodes, with lie trials showing a larger P2 than truth trials [main effect of Veracity; *F*(1,40) = 36.47, *p* < .001, *η* ^2^_g_ = .13, BF_incl_ = 2.32^e+3^]. This effect was further modulated by the assignment of colors to conditions [interaction of Veracity × Color Mapping; *F*(1,40) = 20.90, *p* < .001, *η* ^2^_g_ = .08, BF_incl_ = 567.97]. Follow‐up ANOVAs revealed that the effect of Veracity was significant only for yellow/truth and blue/lie [*F*(1,21) = 53.58, *p* < .001, *η* ^2^_g_ = .26, BF_incl_ = 3.63^e+4^], but not for yellow/lie and blue/truth [*F*(1,21) = 1.25, *p* = .276, *η* ^2^_g_ = .01, BF_incl_ = 0.49]. No further interactions involving the factor Veracity were observed (all *p*s > .724).

Seeing as the posterior N2 effect and the anterior P2 effect occurred almost simultaneously and the effect of Veracity on N2 amplitude was significantly stronger when yellow signaled the need for lying (see above), potential effects of Veracity on P2 amplitude for this color assignment might have been masked by the large N2 effect. To control for that, we additionally determined the P2 when controlling for the N2 by subtracting N2 amplitudes from P2 amplitudes in the respective conditions. This cleaner P2 again yielded a significant main effect of Veracity [*F*(1,40) = 5.11, *p* < .029, *η* ^2^_g_ = .03, BF_incl_ = 0.68], with lie trials showing a larger P2 than truth trials. Again, this effect was further modulated by the assignment of colors to conditions [interaction of Veracity × Color Mapping; *F*(1,40) = 52.91, *p* < .001, *η* ^2^_g_ = .21, BF_incl_ = 4.48^e+6^]. However, follow‐up ANOVAs now revealed that the effect of Veracity was significant for both color assignments; that is, for yellow/truth and blue/lie [*F*(1,21) = 48.95, *p* < .001, *η* ^2^_g_ = .28, BF_incl_ = 2.48^e+4^] as well as for yellow/lie and blue/truth [*F*(1,21) = 12.85, *p* = .002, *η* ^2^_g_ = .13, BF_incl_ = 25.17]. No further interactions involving the factor Veracity were observed (all *p*s > .864).

**FIGURE 4:**
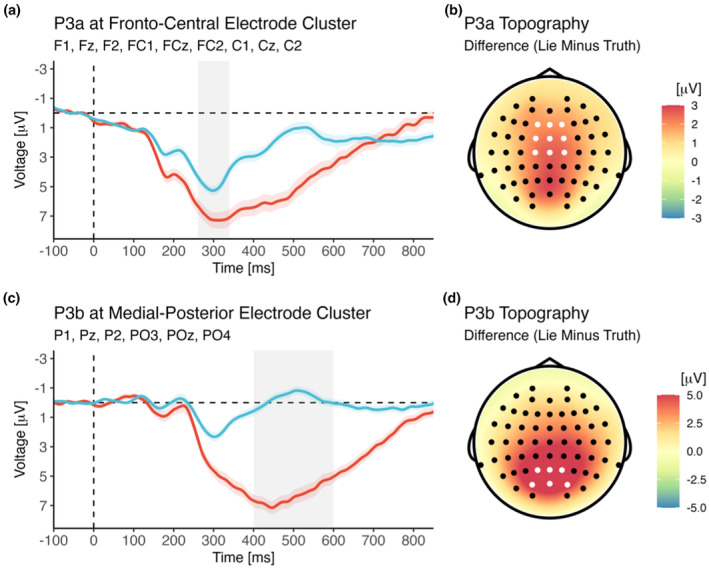
Effect of veracity on ERP amplitudes for late attentional components P3a (a, b) and P3b (c, d). The shaded area marks the time window selected for analysis (see main text for details). Note that the frontal P3a overlapped with the slightly later and more posterior P3b, with the large effect of veracity on P3b amplitude causing a seemingly posterior peak of the P3a difference (between lie and truth trials). However, the P3a itself was largest at frontocentral electrodes when observed separately for lie and truth trials and when regarding the grand‐grand average. Thus, in line with the large body of literature on the P3a, amplitude effects were analyzed at the typical frontocentral electrode cluster.

As shown in Figure 4, P3a amplitude was larger for lie trials than for truth trials [main effect of Veracity; *F*(1,40) = 48.03, *p* < .001, *η* ^2^_g_ = .75, BF_incl_ = 9.97^e+5^]. This effect was not further modulated by the assignment of colors to conditions or the assignment of responses to buttons (all *p*s > .335).

P3b amplitude was notably larger for lie trials than for truth trials [main effect of Veracity; *F*(1,40) = 338.65, *p* < .001, *η* ^2^_g_ = .16, BF_incl_ = 3.92^e+24^]. This effect was further modulated by the assignment of colors to conditions [interaction of Veracity × Color Mapping; *F*(1,40) = 8.74, *p* = .005, *η* ^2^_g_ = .07, BF_incl_ = 10.96] as well as by the assignment of responses to buttons [interaction of Veracity × Button Mapping; *F*(1,40) = 4.41, *p* = .042, *η* ^2^_g_ = .04, BF_incl_ = 1.70]. No further interactions involving the factor Veracity were observed (*p* > .724). Follow‐up ANOVAs revealed that the effect of Veracity was significant for yellow/truth and blue/lie [*F*(1,21) = 189.50, *p* < .001, *η* ^2^_g_ = .79, BF_incl_ = 4.02^e+14^] as well as for yellow/lie and blue/truth [*F*(1,21) = 130.10, *p* < .001, *η* ^2^_g_ = .62, BF_incl_ = 1.14^e+10^]. Likewise, the effect of Veracity was significant for left/furniture and right/clothing [*F*(1,21) = 154.80, *p* < .001, *η* ^2^_g_ = .74, BF_incl_ = 5.25^e+12^] as well as for left/clothing and right/furniture [*F*(1,21) = 143.10, *p* < .001, *η* ^2^_g_ = .73, BF_incl_ = 1.11^e+12^].

**FIGURE 5:**
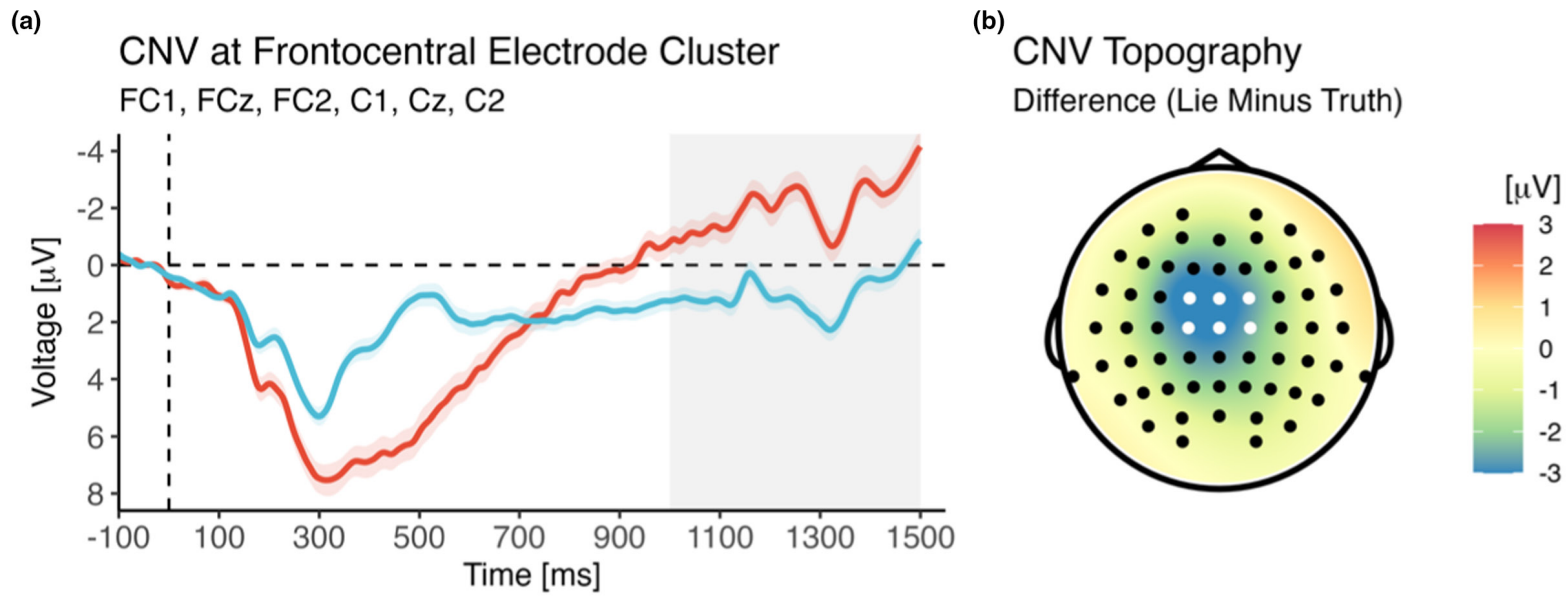
Effect of veracity on the CNV following cue onset and leading up to target onset, shown as voltage over time (a) and as voltage difference between conditions during the time window selected for analysis (b). The shaded area marks the time window selected for analysis (see main text for details).

As shown in Figure 5, CNV amplitude was markedly larger for lie trials than for truth trials [main effect of Veracity; *F*(1,40) = 67.12, *p* < .001, *η* ^2^_g_ = .28, BF_incl_ = 1.19^e+8^]. This effect was not further modulated by the assignment of colors to conditions or the assignment of responses to buttons (all *p*s > .339).

As reported above, the precautionary inclusion of counterbalanced manipulations (button and color mapping) in our analyses revealed that they affected to some extent the impact of our main factor of interest (veracity) on ERP amplitudes during cue processing. However, it should be noted that these factors only slightly modulated the size of the effects, with the overall difference between truth and lie becoming evident (and statistically significant) for all relevant combinations of factors. Thus, the effect of interest (i.e., the influence of veracity) did *not* depend on any specific choice of button mapping or color assignment but rather persisted across conditions.

#### 3.2.3 Alpha power and source localization

**FIGURE 6:**
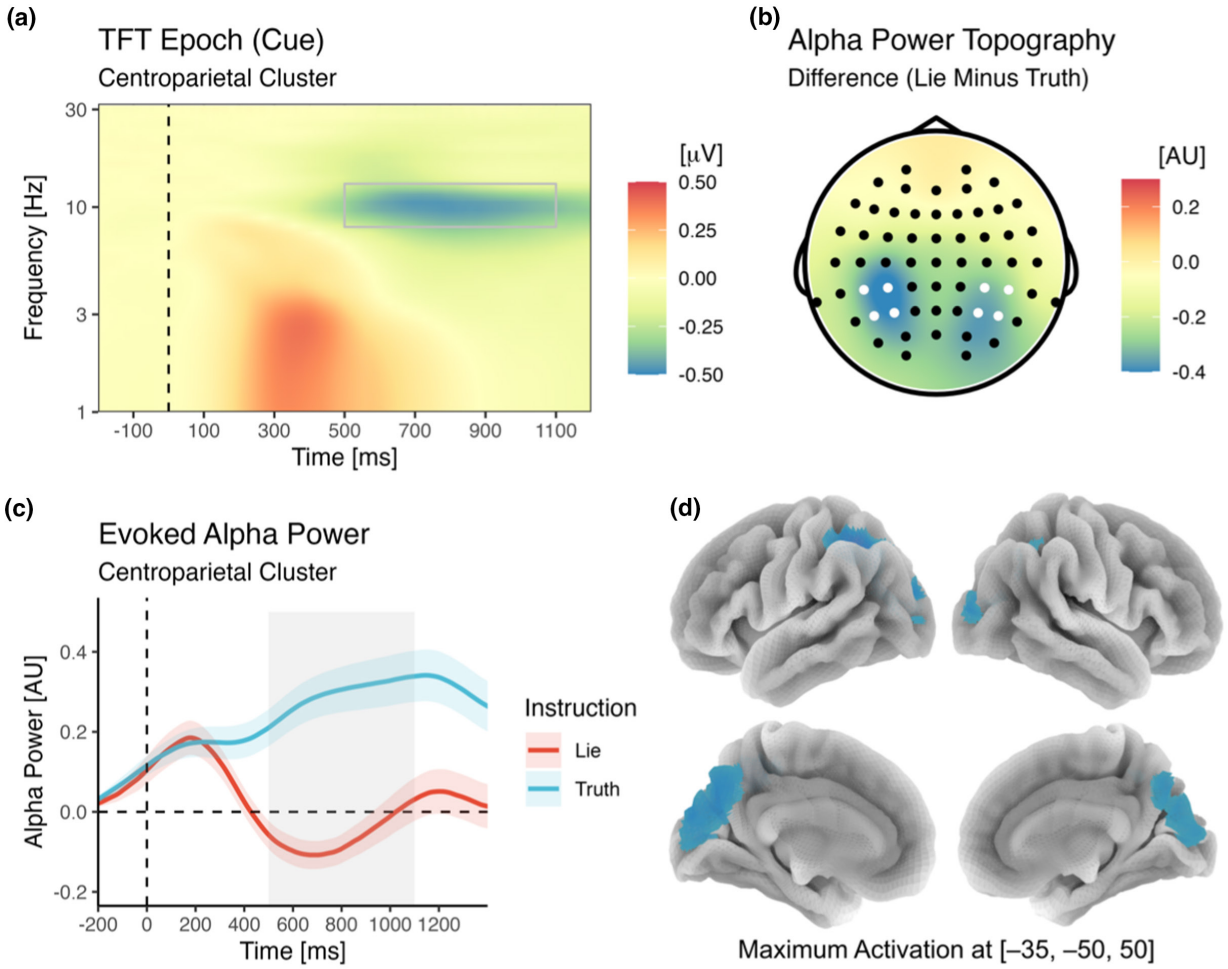
Time‐frequency analysis of cortical oscillations in the alpha band (8–13 Hz) during cue processing. Note that a marked alpha decrease for lie as compared to truth trials was observed between 500 and 1100 ms post‐stimulus (a). This relative alpha suppression was most pronounced at bilateral centroparietal recording sites (b). The averaged difference in centroparietal alpha power was significant (c), and DICS beamforming suggested a source in the SPL in BA7 (d). AU, arbitrary units. Brain coordinates are MNI coordinates.

As shown in Figure 6, for oscillatory activity during cue processing, a significant cluster of decreased power for lie as compared to truth trials was observed in the alpha band between 500 and 1100 ms after stimulus onset. The spatial distribution of this difference was largest at centroparietal sites. Focusing on this location (by averaging power across centroparietal electrodes; see Method section) and this time frame, the difference between lie and truth trials was significant [*F*(1,43) = 116.90, *p* < .001, *η* ^2^_g_ = .25, BF_incl_ = 5.13^e+10^]. Using DICS beamforming, the primary source of this oscillatory difference was estimated to be located in BA7 (approximate MNI coordinates: −35, −50, 50), in the parietal cortex in the area of the precuneus and the superior parietal lobule (SPL).

### 3.3 Target processing

#### 3.3.1 ERPs (N400, LPC)

**FIGURE 7:**
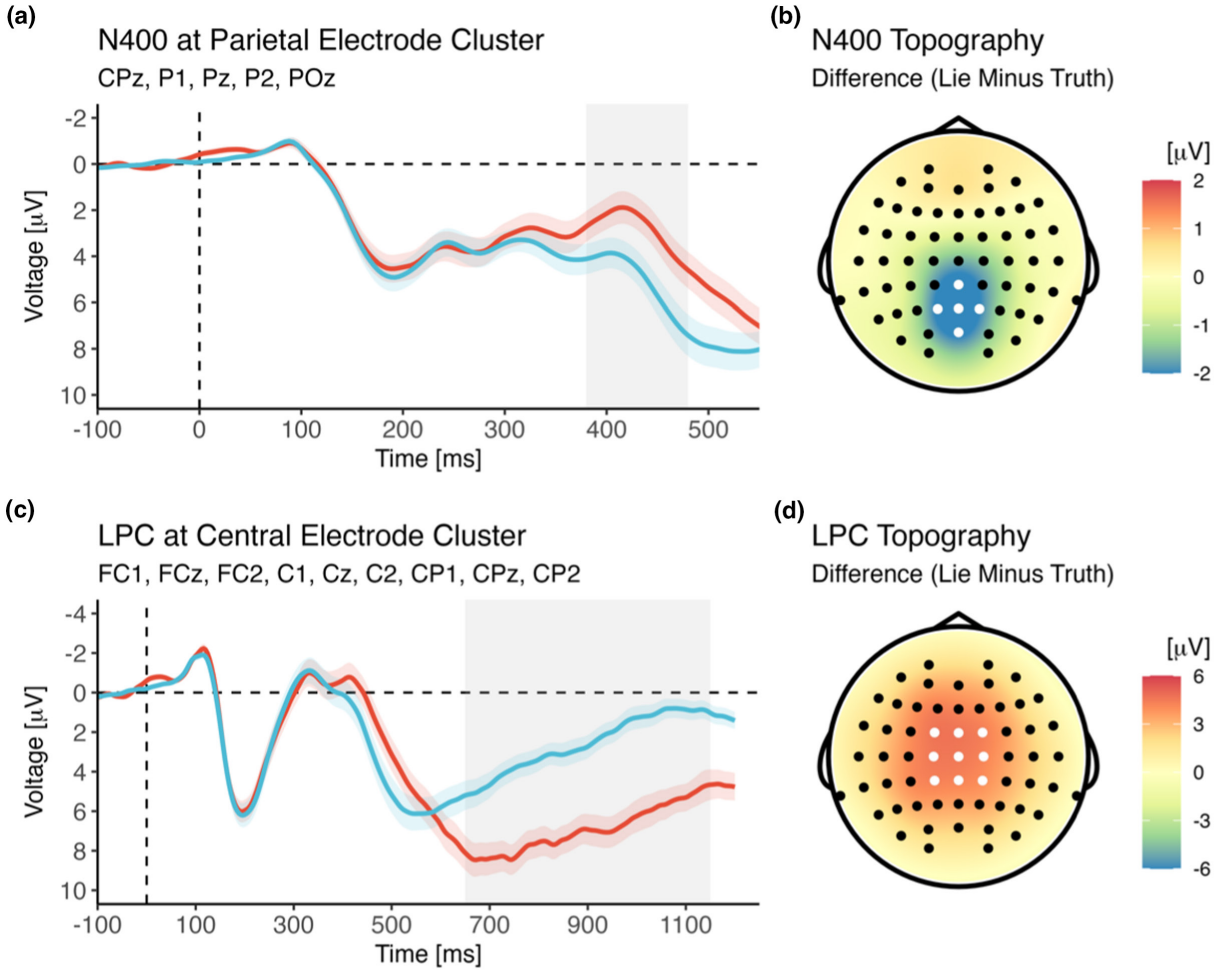
Effect of veracity on N400 and LPC amplitude during target processing, shown as voltage over time (a, c) and as voltage difference between conditions during the time window selected for analysis (b, d). The shaded area marks the time window selected for analysis (see main text for details).

While no stimulus‐locked MFN (or anterior N2) was observed, a slightly later negativity, namely a previously unanticipated N400 difference between lie and truth trials, was observed at central electrodes (see Figure 7), with lie trials showing a larger N400 than truth trials [main effect of Veracity; *F*(1,40) = 50.81, *p* < .001, *η* ^2^_g_ = .06, BF_incl_ = 8.53^e+5^]. This effect was not further modulated by the assignment of colors to conditions or the assignment of responses to buttons (all *p*s > .077).

LPC amplitude was larger for lie trials than for truth trials [main effect of Veracity; *F*(1,40) = 92.43, *p* < .001, *η* ^2^_g_ = .26, BF_incl_ = 2.75^e+9^]. This effect was not further modulated by the assignment of colors to conditions or the assignment of responses to buttons (all *p*s > .058).

#### 3.3.2 Theta power and source localization

**FIGURE 8:**
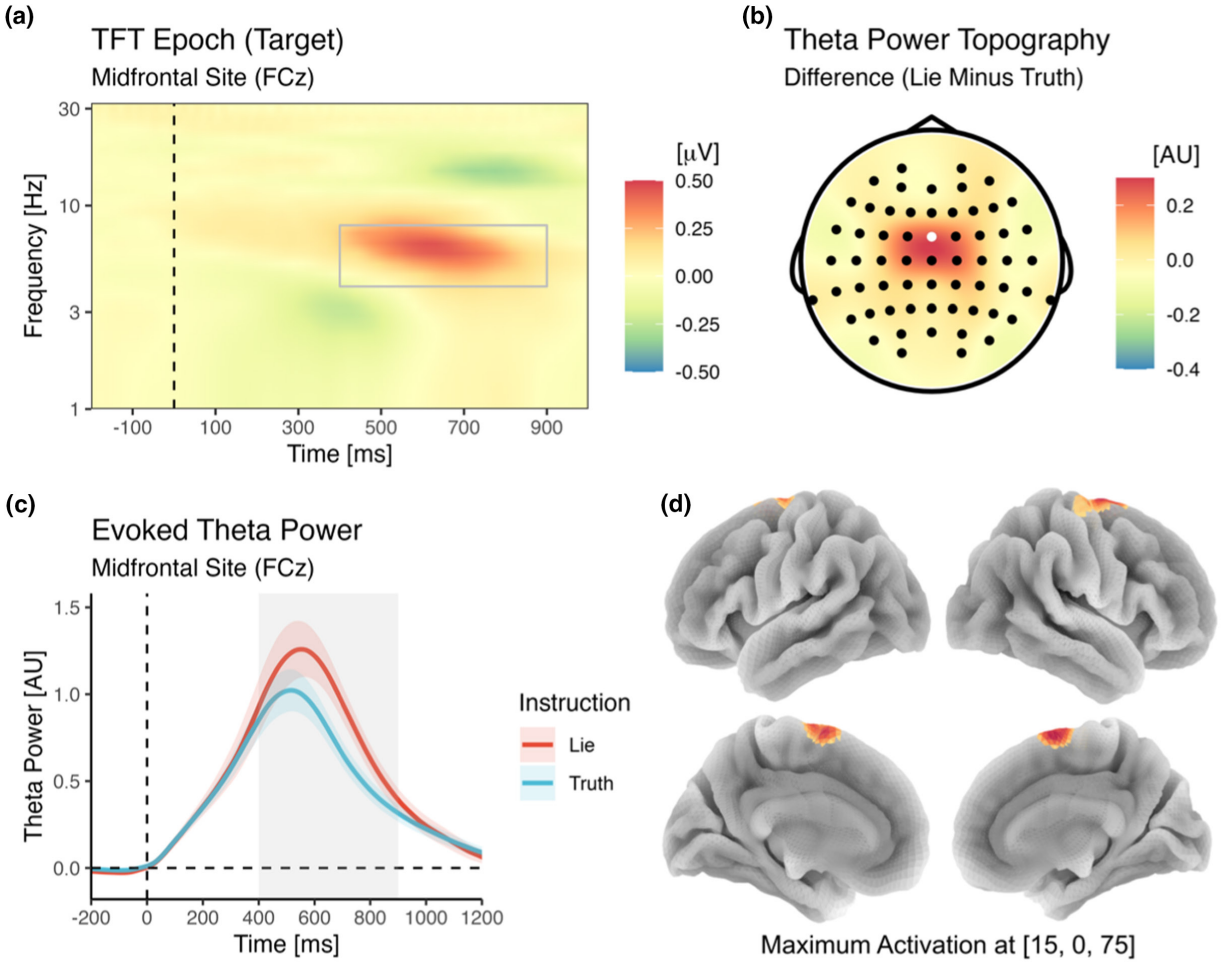
Time‐frequency analysis of cortical oscillations in the theta band (4–8 Hz) during target processing. Note that the expected theta power increase for lie as compared to truth trials was observed between 400 and 900 ms post‐stimulus (a). This relative increase of theta oscillations was most pronounced at midfrontal recording sites (b). The averaged difference in midfrontal theta power was significant (c), and DICS beamforming suggested a source in the SMA in BA6 (d). AU, arbitrary units. Brain coordinates are MNI coordinates.

As shown in Figure 8, for oscillatory activity during target processing, a significant cluster of increased power for lie as compared to truth trials was observed in the theta band between 400 and 900 ms after stimulus onset. The spatial distribution of this difference was most pronounced around electrode FCz. Power at this site and in this time frame was significantly different between lie and truth trials [*F*(1,43) = 9.76, *p* = .003, *η* ^2^_g_ = .03, BF_incl_ = 10.89]. Using DICS beamforming, the primary source of this oscillatory difference was estimated to be located in BA6 (approximate MNI coordinates: 15, 0, 75), in the posterior section of the frontal cortex in the supplementary motor area (SMA).

## 4 DISCUSSION

In the present study, we investigated neurophysiological signatures of basic cognitive processes during the anticipation and preparation as well as the execution of deceptive behavior. Using a straightforward experimental approach involving instructed false responding, we were able to replicate previous findings on increased executive control and attention during the processing of stimuli to which deceptive (as compared to truthful) responses are required. We did, however, also expand these findings by showing that preceding cues indicating the need for subsequent deception elicit a series of additional processes of attention, anticipation, and preparation in the foreperiod leading up to the stimulus requiring a lie.

### 4.1 Novel findings

The need to lie becomes obvious not only when we encounter a stimulus to which we have to respond, but often presents itself earlier, with questions or word sequences leading up to that stimulus signaling in advance the subsequent necessity of lying (for specific examples, see DePaulo et al., 1996). We investigated the neurocognitive processes involved in the analysis of such preceding cues, using ERPs and oscillatory cortical activity to further our understanding of the contributing processes during lying.

First of all, we show that early attentional selection appears to be enhanced when a cue signaling the need for subsequent lying is being processed: Both the posterior N2 and the frontal P2 were increased for lie as compared to truth cues. While we did not make predictions regarding the posterior N2, we explored this interesting finding and offer an explanation based on previous studies on early attentional selection. It should be noted, however, that future studies employing hypothesis‐driven analyses of such early effects in sequential deception paradigms, such as the cue‐based setup in the present study, are necessary to replicate this finding and further understand its nature.

The posterior N2, which has to be distinguished from a conflict‐related anterior N2, most likely reflects early attentional prioritization during visual processing and is highly sensitive to stimulus frequency (Potts, 2004). Fittingly, cues indicating the subsequent need for lying, naturally occurring less frequently than honesty cues, are processed differently already at such early stages of visual processing, with neurons presumably responding more strongly to rare (and, thus, more relevant) stimuli as compared to the previously established “neural pattern” triggered by more frequent visual events (Nieuwenhuis et al., 2003; Potts et al., 1996). This mismatch coding presumably takes place along the frontoparietal attention network (Bocquillon et al., 2014; Crottaz‐Herbette & Menon, 2006; see also Bocquillon et al., 2011). Importantly, this effect was shown for both assignments of colors to conditions (lie vs. truth being signaled by yellow vs. blue cues), thus precluding the possibility that early posterior effects merely reflect differences between lie and truth cues in terms of physical stimulus attributes.

The frontocentral P2 effect of veracity could be interpreted in a similar way as the N2 effect: During early visual processing, attention toward relevant events increases (Kosonogov et al., 2019; Lu et al., 2016; Wang et al., 2021; for P2 in instructed deception, see Gibbons et al., 2018). At first glance, we observed this effect only when the rare lie cue was blue (and the standard truth cue was yellow), but not when the assignment of colors to conditions was reversed. However, the almost simultaneously occurring posterior N2 effect showed the reverse pattern, with the effect of veracity being larger when the lie cue was yellow than when it was blue. Therefore, the N2 effect at posterior sensors could have reduced the frontal P2 effect, with the very large N2 effect for yellow lie cues canceling out the analogous P2 effect. When controlling for N2 amplitude, we indeed found significant effects of veracity (lie vs. truth) on P2 amplitude for both variants of color assignments. P2 amplitude was increased for the cue indicating the need for subsequent lying as compared to it signaling the need for subsequent truth‐telling. This supports the idea that early attentional selection is enhanced when a stimulus conveys the need for subsequent deception (see also Gibbons et al., 2018).

Increased attention to the cue when it signaled the need for lying (as compared to the need for honesty) was also observed at later stages, with both the frontal P3a and a more posterior, broadly distributed P3b being significantly larger for lie than for truth cues. It has previously been shown that encountering a response‐inducing stimulus to which one has to respond deceptively involves signatures of greater task relevance (P3a) as well as of greater in‐depth attentional processing (P3b) of words perceived to be the “targets” among the standard stimuli demanding honest responses (Gibbons et al., 2018). Our findings complement this idea by showing that a very similar process occurs for cues that are not response‐relevant per se, but rather convey the need for lying in response to a later target. Interestingly, learning about the need to lie in the present paradigm does not involve the possibility to prepare a specific response (e.g., a left‐hand button press); after all, as long as the imperative stimuli has not been shown, participants cannot tell whether a lie would be a left‐hand or right‐hand response. Therefore, the early (N2, P2) and later (P3a, P3b) attentional effects for lying (as compared to truth‐telling) cues neatly demonstrate that such enhancements are not directly linked to the evaluation of the imperative stimulus and the selection or preparation of the appropriate response. Instead, they reflect purely *cognitive* processes triggered by the rare, relevant lying cues.

We also tested whether knowing that the subsequent stimulus will require a deceptive (rather than truthful) response results in altered processes shortly before the actual imperative stimulus appears. To this end, we investigated the CNV and alpha oscillations in the foreperiod following the cue and leading up to the target. CNV amplitude was markedly larger after cues indicating the need for lying than for those indicating the need for truthful responses. This suggests that lying cues lead to anticipation and preparation, potentially at the perceptual, cognitive, and motor level (Anatürk & Jentzsch, 2015; Bauer et al., 1992; De Kleine & Van Der Lubbe, 2011; Getzmann et al., 2016; Hackley et al., 2014; Kóbor et al., 2021; Pauletti et al., 2014; Stadler et al., 2006; Wild‐Wall et al., 2007; Wöstmann et al., 2015). Fittingly, we also found that during this foreperiod, alpha power, a measure of idle activity of the brain, is suppressed in parietal brain areas (peaking in BA7) linked to sensorimotor integration (Gamberini et al., 2021), goal‐directed attention (Corbetta & Shulman, 2002; Molenberghs et al., 2007), and cognitive flexibility during task switching (Wendiggensen & Beste, 2023). It has been suggested that BA7 involvement occurs during executive control “whenever incoming information is complex and probably difficult to categorize but essential for behavioral control” (Petruo et al., 2019, p. 112; see also Bodmer & Beste, 2017; Ocklenburg et al., 2011). Taken together, CNV amplitudes and alpha oscillations concordantly suggest that our cognitive system is preparing for upcoming lies, with brain structures subserving attentive, controlled, and purposeful actions remaining particularly excitable instead of returning to a baseline level of highly synchronized alpha activity. Such preparatory processes seem highly adaptive in light of the fact that lying represents a deviation from default behavior (Verschuere & Shalvi, 2014) and is cognitively demanding (Suchotzki et al., 2017).

### 4.2 Replication and extension of previous findings

We additionally set out to replicate the well‐established finding that conflict monitoring and resolution, as indicated by the MFN, increase during the processing of stimuli requiring deceptive as compared to those requiring truthful responses (e.g., Gibbons et al., 2018; Hu et al., 2011; Scheuble & Beauducel, 2020; Suchotzki et al., 2015). Interestingly, we did observe an increased negativity for lie (as compared to truth) targets; however, it was not a frontal component (such as the conflict‐related anterior N2 or the response‐accompanying MFN), but rather a posterior effect in the N400 time range (Kutas & Hillyard, 1980). It has previously been shown that situations of socially relevant conflict, usually believed to involve early processes of executive control as indicated by MFN‐like components, result in N400 effects instead (Huang et al., 2014; Schnuerch et al., 2016). Crucially, the N400 has been proposed to reflect not only semantic incongruity and the ensuing need for integration (Kutas & Federmeier, 2011), but also late semantic inhibition processes (Debruille, 2007). Information that was activated, but is currently inappropriate, might be inhibited (Sinha et al., 2023). In a similar vein, it might be an index of highly controlled semantic processing (Chwilla et al., 1995; Pires et al., 2014). This is well in line with the notion that deception involves a high level of executive control (Abe, 2009), including the management of a predominant association (in the present study: the category to which the word actually belongs) that has to be suppressed in order to respond in a semantically false way (in the present study: falsely classifying the word as belonging to a different semantic category).

Why previous studies have observed such processes in the N2 and/or MFN time rage and at frontocentral sites, while we have found an electrophysiological signature of semantic conflict detection and inhibitory processes around 400 ms and at central (to posterior) sites, is yet unclear. The most obvious difference between previous studies and the present one is the fact that we included a cue that *precedes* the target stimulus (rather than the target stimulus itself or a concurrent color informing about the need to lie). As reported above, this leads to several cognitive processes changing already during cue processing and in the period between cue and target. Due to this shift of cognitive operations subserving successful deception to a period before the imperative stimulus, target processing might involve slightly different processes during lying than when both the target itself and the need for lying are perceived simultaneously. First, a preceding cue might lead to an attenuation (or even removal) of the typical motor response conflict observed during lying, seeing as participants in our study already knew (based on the cue) *before* the onset of the imperative stimulus that the motor response triggered by the target word must not be transformed into actual behavior. They could thus remap the categories to response buttons following each lie cue, only triggering (without a proper motor response conflict) the necessary button press after having evaluated the target. Second, the need for lying must be kept in mind from cue onset through target processing, even though the current condition (lie vs. truth) is no longer visible. Thus, conflict monitoring and resolution involves *retrieving* the need for inhibition from working memory and intentionally false responding rather than directly deriving this need from the target itself (Gibbons et al., 2018) or from a simultaneous cue (Suchotzki et al., 2015). However, future studies are clearly needed to discern whether and how preceding cues delivering the need for lying alter cognitive control processes triggered by the actual response‐inducing targets.

To assess cognitive control in a different way, we also analyzed theta oscillations during target processing. This yielded a clear effect of veracity, with targets requiring deceptive responses involving a marked increase in midfrontal theta power as compared to targets requiring truthful responses. This is converging evidence of the enhanced level of executive control, most likely involving conflict detection and resolution, when a stimulus entails a well‐associated semantic evaluation (e.g., a jacket clearly being a piece of clothing), which must be overridden in order to cover the truth (e.g., by classifying the jacket as a piece of furniture). Our source analysis suggests that this effect involves activity in the SMA, which has previously been implicated in cognitive control (Sjöberg et al., 2019; see also Irlbacher et al., 2014). Interestingly, similar to the ERP effect (N400), the oscillatory effect of veracity (theta increase) was observed in a time frame that does not coincide with the typical conflict‐related N2 or stimulus‐locked MFN (around 300 ms), but slightly later (400–900 ms). Thus, both types of measurement (ERPs and cortical oscillations) congruently indicate a relatively late point in time at which cognitive control occurs. While one might have expected previous preparation to speed up (rather than to slow down) the sequence of involved processes, the mere involvement of a preceding cue might have shifted the target‐induced cognitive operations involved in lying. Again, however, this remains speculative and should be investigated in more detail in future studies.

Finally, we replicated previous findings showing that the processing of target stimuli to which deceptive (as compared to truthful) responses have to be given is characterized by an increased allocation of sustained attentional resources, as indicated by an enhanced posterior positivity such as the LPC (Gibbons et al., 2018). Several studies, however, have reported that deception (as compared to truthful behavior) was accompanied by a reduced (rather than increased) LPC/P3b amplitude. All of these studies employed equal proportions of both types of events (Hu et al., 2011; Pfister et al., 2014; Suchotzki et al., 2015; Wu et al., 2009), and the reduced P3 in this context might indicate higher workload during lying as compared to truth‐telling, thus reflecting “the dual task character of lying: lying requires the truth to be kept active, monitored and at the same time inhibited” (Suchotzki et al., 2015, p. 400). In our study, as well as in similar previous investigations on deception and concealed information (Gibbons et al., 2018; see also Verschuere et al., 2009), lie trials were less frequent than truth trials. Therefore, the enhanced LPC reflects, at least partly, the attentional response to a rare, and thus, within the given context, particularly relevant event (Suchotzki et al., 2015; see also Gable & Harmon‐Jones, 2010; Ito et al., 1998; Hajcak et al., 2007; Ziereis & Schacht, 2023). Greater LPC has also been implicated in the strategic adaptation of behavior on subsequent trials (e.g., following error feedback; Gibbons et al., 2016); in the present context, increased strategic adaption might be triggered by the occurrence of a (rare, more demanding) deception trial. However, this is not a contradiction to the processes involved in lying, seeing as deception actually occurs less frequently in real life (Verschuere & Shalvi, 2014). This finding thus captures the fact that lies by their very nature as infrequent types of behavior, involve an increased allocation of cognitive resources during the evaluation of the rare stimulus to which dishonest responses are required.

### 4.3 Limitations

Clearly, the present study is merely an approximation to capturing the neurocognitive processes involved in human deceptive behavior. As such, there are several limitations. First, our sample was biased toward young, female, student participants, which may reduce the generalizability of the results. Second, to capture lying under laboratory conditions, participants are typically explicitly instructed to do so (Spence et al., 2001), thus excluding any intrinsic motives for deception as well as any naturalistic emotional subprocesses (see Abe, 2011). Third, we did not test for the actual causal contribution of neurocognitive processes to the successful act of lying. That is, we cannot claim which process actually enables us to deceive or which neural computation is necessary for deception. Fourth, an experimental protocol in which lies are relatively rare makes trials requiring participants to deceive stand out (from the majority of trials requiring truthful responses). Thus, lying appears as a sort of target behavior, most likely entailing an unspecific, oddball‐driven allocation of attention to stimuli signaling the need for lying. This increase in selective attention, however, does not represent any deception‐inherent process per se, but might mostly reflect the infrequency or target status of lies in the experimental setup (see Verschuere et al., 2011). Future studies might rule out the contribution of relative event frequency to the neural signatures of lie (vs. truth) trials by making both types of events equally likely. It should be noted, however, that it is reasonable to assume that, in real life, lying is less frequent than truth‐telling (see Verschuere & Shalvi, 2014), such that any oddball‐like attentional capture of rare events requiring deception still represents a typical (albeit unspecific) cognitive process involved in the act of lying (Gibbons et al., 2018).

## 5 CONCLUSION

Using both ERPs and analyses of cortical oscillations, we have replicated and extended previous findings on the neurocognitive processes involved in deception. Our experimental setup clearly excludes affective and motivational aspects of lying, isolating only the cognitive processes by means of instructing participants to lie somewhat artificially and reactively (rather than on their own terms). This is a notable limitation common to many experimental approaches in this field (e.g., Hu et al., 2011, 2012; Spence et al., 2001; Suchotzki et al., 2015; Suchotzki & Gamer, 2018). However, it still captures the essence of the mental operations necessary to successfully deceive by responding in the semantically wrong way (Suchotzki et al., 2017). Here, we show that lying can begin as early as a few hundred milliseconds into a cue that lets us know that we subsequently have to lie, with early and late attentional selection as well as anticipatory and preparatory processes increasing well before the target in response to which we have to lie. In our view, this finding could have the potential to stimulate the development of new and innovative approaches to lie detection. Additionally, we replicate and extend to the level of cortical power oscillations previous findings that the processing of the stimulus to which we respond deceptively involves increased executive control (conflict processing and/or inhibition) as well as sustained, motivated attention.

## AUTHOR CONTRIBUTIONS

**Robert Schnuerch:** Conceptualization; formal analysis; investigation; project administration; visualization; writing – original draft; writing – review and editing. **Jonas Schmuck:** Data curation; formal analysis; investigation; methodology; resources; visualization. **Henning Gibbons:** Conceptualization; funding acquisition; resources; supervision; validation; writing – review and editing.

## FUNDING INFORMATION

None.

## CONFLICT OF INTEREST STATEMENT

We declare that there is no conflict of interest.

## Data Availability

The data that support the findings of this study are available from the corresponding author, Robert Schnuerch, upon reasonable request.
